# Cytochrome P450 Monooxygenases *CYP6AY3* and *CYP6CW1* Regulate Rice Black-Streaked Dwarf Virus Replication in *Laodelphax striatellus* (Fallén)

**DOI:** 10.3390/v13081576

**Published:** 2021-08-10

**Authors:** Jian-Hua Zhang, Ming Zhao, Yi-Jun Zhou, Qiu-Fang Xu, Yuan-Xue Yang

**Affiliations:** 1 Key Laboratory of Food Quality and Safety of Jiangsu Province, State Key Laboratory Breeding Base, Institute of Plant Protection, Jiangsu Academy of Agricultural Sciences, Nanjing 210014, China; zhangjianhua198904@163.com (J.-H.Z.); yjzhou@jaas.ac.cn (Y.-J.Z.); 2 Institute of Industrial Crops, Shandong Academy of Agricultural Sciences, Jinan 250100, China; scrczhm@163.com; 3 Institute of Life Sciences, Jiangsu University, Zhenjiang 212013, China

**Keywords:** insecticides, *Laodelphax striatellus*, rice black-streaked dwarf virus, P450s, *CYP6CW1*, *CYP6AY3*, replication

## Abstract

The small brown planthopper, *Laodelphax striatellus* (Fallén), is an important agricultural pest that causes significant losses by sucking and transmitting multiple plant viruses, such as rice black-streaked dwarf virus (RBSDV). Insecticides are commonly used to control planthoppers and cause the induction or overexpression of cytochrome P450 monooxygenases (P450s) from the CYP3 and CYP4 clades after insecticide application. However, little is known about the roles of insecticides and P450s in the regulation of viral replication in insects. In this study, RBSDV-infected *L. striatellus* were injected with imidacloprid, deltamethrin, pymetrozine, and buprofezin, respectively. The insecticide treatments caused a significant decrease in RBSDV abundance in *L. striatellus*. Treatment of piperonyl butoxide (PBO), an effective inhibitor of P450s, significantly increased the RBSDV abundance in *L. striatellus*. Fourteen P450 candidate genes in the CYP3 clade and 21 in the CYP4 clade were systematically identified in *L. striatellus*, and their expression patterns were analyzed under RBSDV infection, in different tissues, and at different developmental stages. Among the thirty-five P450 genes, the expression level of *CYP6CW1* was the highest, while *CYP6AY3* was the lowest after RBSDV infection. Knockdown of *CYP6CW1* and *CYP6AY3* significantly increased the virus abundance and promoted virus replication in *L. striatellus*. Overall, our data reveal that *CYP6CW1* and *CYP6AY3* play a critical role in the regulation of virus replication in *L.* *striatellus*.

## 1. Introduction

Rice production is severely affected by planthoppers, such as *Laodelphax striatellus*, *Sogatella furcifera*, and *Nilaparvata lugens*. The rice planthoppers can cause losses by feeding. Besides, they also transmit a variety of plant viruses, causing severe yield losses in rice-producing regions. For example, *S. furcifera* can transmit Southern rice black-streaked dwarf virus, while *N. lugens* can transmit rice grassy stunt virus and rice ragged stunt virus [1,2]. *Laodelphax striatellus* can transmit rice stripe virus, rice black-streaked dwarf virus (RBSDV), maize rough dwarf virus, northern cereal mosaic virus, and barley yellow striate mosaic virus [3,4,5,6,7]. The outbreak of these viral diseases caused severe yield losses in rice-producing regions [4]. Insecticides are widely used to control planthoppers. However, little is known about the effect of insecticide on the virus transmitted by the planthoppers.

Cytochrome P450 monooxygenases (P450) are a group of heme-thiolate enzymes that catalyze a variety of reactions related to metabolism and chemical toxicity [8]. The P450s are divided into CYP2, CYP3, CYP4, and mitochondrial CYP clades. A series of P450s in CYP3 and CYP4 clades are induced or overexpressed in planthoppers and involved in the metabolism of insecticides, such as *CYP4CE1*, *CYP6AY1,* and *CYP6CW1* [9,10,11]. Metabolic resistance mediated by the P450s has been considered as the dominant factor for insecticide resistance in the field populations of planthoppers [12,13,14]. A previous study showed that the genetic variants of *CYP2E1* and *CYP1A1* resulted in the susceptibility to chronic Hepatitis B virus infection [15], suggesting P450s in CYP3 and CYP4 clades might be involved in the regulation of virus infection.

Rice black-streaked dwarf virus is one of the important viruses transmitted by *L. striatellus*. It is the causal agent of rice black-streaked dwarf and maize rough dwarf disease, which cause considerable yield losses in East Asia [16]. RBSDV belongs to the genus *Fijivirus* of the family *Reoviridae*. Its genome includes ten double-stranded RNA (dsRNA) segments (*S1* to *S10*) and encodes thirteen proteins. Each segment of *S5*, *S7*, and *S9* encodes two proteins. P5-1, P6, and P9-1 proteins, which are encoded by the segments of *S5-1*, *S6*, and *S9-1*, are the components of the viroplasm [17,18,19]. RBSDV P10 protein, encoded by *S10* segment, is the outer capsid protein, which plays a key role in viral infection process [20,21]. Midgut of *L. striatellus* is a barrier for RBSDV infection [22]. Currently, there are no rice cultivars resistant to RBSDV. Using insecticide to control *L. striatellus* population is still one of the important strategies to control the viral disease. High-efficiency, low-toxicity, and low-residue insecticides, such as mesoionic insecticide, triazinone insecticide, pyrethroid insecticide, neonicotinoid insecticide, and insect growth regulator, are most widely used to control planthoppers in the fields [23]. Imidacloprid is a representative neonicotinoid insecticide and can disturb the nervous system of insects [10,11], while deltamethrin is a modulator of nerve sodium channels in insects [24]. Pymetrozine is a fast-acting and selective inhibitor of the feeding of sap-sucking insects [14]. Buprofezin is an insect growth regulator and can interfere with metabolism and inhibit chitin synthesis in insects [14]. Fourteen P450s were overexpressed in the deltamethrin-resistant *L. striatellus* strain [24]. Overexpression of *CYP6CW1* was involved in the resistance to buprofezin and pymetrozine in *L. striatellus* populations [14]. Imidacloprid treatment significantly upregulates the P450 gene *CYP4C71* in *L. striatellus* [25]. The function of P450s in metabolism of insecticides in insects is well studied. However, whether P450 genes play a role in regulation of virus replication in *L. striatellus* is largely unknown.

In this study, the effect of insecticides on viruses was investigated using RBSDV and *L. striatellus* interaction system. Four representative insecticides, including imidacloprid, deltamethrin, pymetrozine, and buprofezin, were selected to study whether insecticides affect RBSDV infection in *L. striatellus*. We showed that the application of these four insecticides reduced the RBSDV abundance in *L. striatellus*. Furthermore, inhibition of the P450s activities by P450s inhibitor piperonyl butoxide (PBO) significantly decreased the RBSDV abundance in *L. striatellus*. Knockdown of P450s *CYP6AY3* and *CYP6CW1* negatively regulated the RBSDV abundance and replication in *L. striatellus*. These results demonstrated that insecticides and P450s can regulate virus infection in the insects. 

## 2. Materials and Methods

### 2.1. Insects

The virus-free (VF) populations of *L. striatellus* were collected from Haian (32.57° N, 120.45° E; Jiangsu, China) in 2004 and maintained in the laboratory. The *L. striatellus* was reared on rice seedlings at 25 ± 1 °C with 70−80% humidity and a photoperiod of 16 h light/8 h dark in a growth chamber. The 3rd-instar VF nymphs of *L. striatellus* were reared on RBSDV-infected (RB) rice plants for two days. After rearing on healthy rice seedlings for another three days, the nymphs were collected as RB *L. striatellus*. 

### 2.2. Quantitative Real-Time PCR (RT-qPCR) 

RT-qPCR was adopted to analyze the relative gene expression as previously described [26]. Total RNA was extracted by TRIzol reagent (Invitrogen, Waltham, MA, USA). The cDNA was synthesized from 1 μg of total RNA using PrimeScript^TM^ RT reagent kit with gDNA Eraser (Takara, Kusatsu, Japan) according to the manufacturer’s instruction. Quantitative PCR reactions were conducted on iQ5 real-time PCR system (Bio-Rad, Hercules, CA, USA) using SYBR PrimeScript^TM^ RT-PCR Kit (Takara, Kusatsu, Japan). Each PCR reaction included three independent technical and biological replications. Ribosomal protein L5 (*RPL5*) was used as an internal reference gene [26]. The relative expression levels of target genes were calculated by 2^−ΔΔCt^ [26]. The primers with a product length of 110–160 bp were designed by Beacon Designer 7.7, and the details of the primers are listed in Supplemetary Materials, Table S1. 

### 2.3. RNA Interference (RNAi)

For the RNAi assay, partial sequences of *CYP6CW1*, *CYP6AY3,* and enhanced green fluorescent protein (EGFP) were amplified by PCR using the special primers (Table S1). Then, the ds*CYP6CW1*, ds*CYP6AY3,* and ds*EGFP* were synthesized using the T7 high yield transcription kit (Invitrogen, Waltham, MA, USA) according to the manufacturer’s instructions. The VF 3rd-instar nymphs were collected and injected with 100 nL ds*CYP6CW1* and ds*CYP6AY3* (2 μg/μL) by a FemtoJet microinjector (Eppendorf, Hamburg, Germany), respectively. Meanwhile, the VF 3rd-instar nymphs were injected with the ds*EGFP* as a control. The RNAi efficiency was determined by calculating the relative expression levels of *CYP6CW1* and *CYP6AY3* at one, three, five, and seven days after dsRNA injection. 

To analyze the effect of *CYP6CW1* and *CYP6AY3* on virus abundance and replication, the VF 3rd-instar nymphs were reared on RB rice plants for two days and, then, collected for injection with 100 nL ds*CYP6CW1* and ds*CYP6AY3* (2 μg/μL) by FemtoJet microinjector (Eppendorf, Hamburg, Germany), respectively. The RB nymphs were injected with the ds*EGFP* as a control. After injection, the nymphs were transferred to healthy rice seedlings for another three days and collected for RT-qPCR and immunofluorescence assay. The experiments were repeated three times independently, and each sample contained thirty insects.

### 2.4. Insecticide Treatment

The insecticides technical of imidacloprid (96.70%), deltamethrin (98.00%), pymetrozine (98.00%), and buprofezin (97.50%) were purchased from Sigma-Aldrich (St. Louis, MO, USA). The four insecticides were dissolved in dimethyl sulfoxide (DMSO) and diluted to 3.50 mg/L, 1.00 mg/L, 9.27 mg/L, and 1.35 mg/L in acetone according to LD_50_ or LC_50_. The VF 3rd-instar nymphs were reared on RB rice seedlings for two days and collected as RB *L. striatellus*. Each insecticide (100 nL) was injected into thirty RB nymphs in the conjunction between prothorax and mesothorax by FemtoJet microinjector (Eppendorf, Hamburg, Germany). Acetone was injected as a control. The injected nymphs were transferred to healthy rice seedlings for three days. The effects of insecticides on virus abundance were determined by changes in the relative expression levels of *S5-1*, *S6*, *S9-1*, and *S10* by RT-qPCR. The RT-qPCR experiments were conducted on a iQ5 real-time PCR system (Bio-Rad, Hercules, CA, USA) using SYBR PrimeScript^TM^ RT-PCR Kit (Takara, Kusatsu, Japan). The experiments were repeated three times independently, and each sample contained thirty insects.

### 2.5. Treatment of P450 Inhibitor PBO 

The 3rd-instar VF nymphs of *L. striatellus* were reared on RB rice plants for two days and injected with 100 nL PBO (20 μg/μL, Sigma-Aldrich, St. Louis, MO, USA) using a FemtoJet microinjector (Eppendorf, Hamburg, Germany). Acetone was injected as a control. The injected nymphs were transferred to healthy rice seedlings for three days. Thirty nymphs were collected for each sample. The RBSDV abundance in *L. striatellus* was determined by analyzing the expression of *S10* using RT-qPCR and labeling the P10 protein in vivo by immunofluorescence. Each sample contained thirty nymphs, and the experiment was repeated three times.

### 2.6. Identification of P450 Genes in CYP3 and CYP4 Clades 

The potential sequences of P450s were obtained from the *L. striatellus* genome (http://dx.doi.org/10.5524/100361 (accessed on 20 July 2019); BioProject: PRJNA393384, PRJNA398597) [27]. We firstly removed the short (<300 bp) and repeated the sequences manually. The remaining sequences were aligned in the NCBI database (http://www.ncbi.nlm.nih.gov/BLAST (accessed on 1 August 2019)) by BLASTx (*E*-value < 10^−5^). The redundant transcript fragments and faulty annotated sequences were also removed. All of the identified sequences of P450s were further annotated and classified by blast against the non-redundant (Nr) protein database (http://www.ncbi.nlm.nih.gov/ (accessed on 1 August 2019)). The CYP3 and CYP4 clades of identified P450s were obtained according to the classification of insect P450s [12]. 

### 2.7. Preparation of Different Tissues and L. striatellus at Different Developmental Stages

The tissues of midgut, fat body, ovary, and salivary gland of *L. striatellus* were dissected from VF adult. The *L. striatellus* were rinsed three times with 75% ethanol and, then, washed three times with sterilized-deionized water. The tissues were dissected in chilled 1× phosphate-buffered saline (1× PBS, pH7.4) under a stereomicroscope using sterile forceps. Each tissue sample was dissected from fifty VF *L. striatellus*. The 1st-instar to 5th-instar nymphs, male and female adults, were collected from VF *L. striatellus*. The relative expression of P450 genes at the mRNA level in different tissues and different developmental stages was detected by RT-qPCR method. The experiments were repeated three times independently, and each sample contained thirty insects.

### 2.8. Analyzing the Effect of RBSDV Infection on P450 Genes Expression

To analyze the effect of RBSDV infection on P450s expression, VF and RB L. striatellus were prepared. The VF 3rd-instar nymphs of *L. striatellus* were reared on RB rice plants for two days and, then, transferred to healthy rice seedlings until 5th-instar nymphs. The surviving 5th-instar nymphs were collected as RB *L. striatellus* samples and used to analyze the expression of P450 genes under RBSDV infection. The VF nymphs reared on healthy rice seedlings at the same time were collected as VF control. RT-qPCR was performed to detect the relative expression of P450 genes at the mRNA level. Each sample contained thirty nymphs, and the experiment was repeated three times. 

### 2.9. Immunostaining 

The effect of PBO, *CYP6CW1*, and *CYP6AY3* on RBSDV abundance in *L. striatellus* midgut was determined by labeling the P10 protein by immunofluorescence. The effect of *CYP6CW1* and *CYP6AY3* on RBSDV replication in *L. striatellus* midgut was determined by labeling the dsRNA with an anti-dsRNA (J2) antibody and the virus viroplasm component P9-1 protein by immunofluorescence. The RB midgut of *L. striatellus* was dissected and fixed in 4% paraformaldehyde for 1 h. Midguts were permeabilized using 2% Triton X-100 for 30 min and blocked in 3% BSA for 2 h. Then, anti-RBSDV P10 mAb conjugated with FITC was incubated with the samples to label the RBSDV. To label P9-1 and dsRNAs in the midgut, the samples were incubated with the primary antibody of P9-1 pAb and J2 mAb overnight at 4 °C, respectively, and subsequently incubated with fluorescence secondary antibody goat anti-Rabbit TRITC (Invitrogen, Waltham, MA, USA) and goat anti-Mouse Alexa Fluor Plus 405 (Invitrogen, Waltham, MA, USA) for 45 min at room temperature. The actin filaments were labeled by phalloidin (Solarbio, Beijing, China). The confocal images were taken by an LSM 710 (ZEISS, Jena, Germany) confocal microscope. 

### 2.10. Data Analysis 

Statistical analyses were performed by SPSS 20.0 software (IBM Corporation, Armonk, NY, USA). The differences between treatments were compared using the student’s *t*-test. One-way analysis of variance (ANOVA) was used to analyze the expression abundance of selected genes in different tissues and different developmental stages. Data were shown as mean ± standard error (SE) from at least three independent experiments. The *p* values < 0.05 and 0.01 were considered statistically significant and very significant differences, respectively. Different letters indicated significant differences at *p* value < 0.05. The ANOVA F statistic is listed in Table S2. 

## 3. Results

### 3.1. Application of Four Insecticides Inhibits RBSDV Abundance in L. striatellus

**Figure 1:**
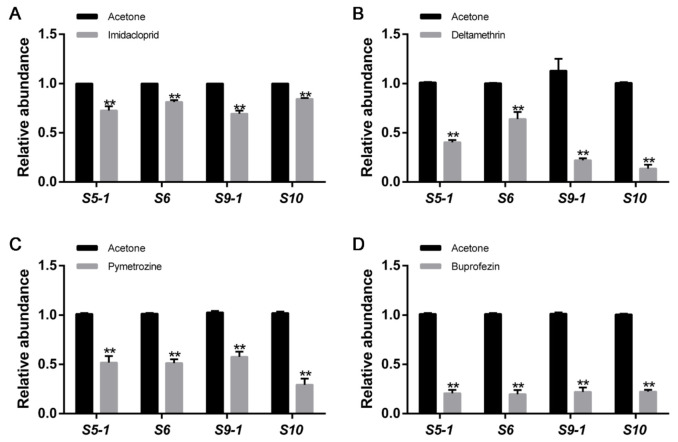
The effects of insecticides on RBSDV infection in *L. striatellus*. (**A**–**D**) The relative expression of RBSDV replication related genes, *S5-1*, *S6,* and *S9-1,* and coat protein gene, *S10*, were significantly decreased in *L. striatellus* after injection of imidacloprid, deltamethrin, pymetrozine, and buprofezin by microinjection, respectively. The asterisks (**) indicate *p* < 0.01.

To investigate the effect of the insecticides on virus infection in the insect, four representative insecticides, including neonicotinoid insecticide imidacloprid, pyrethroid insecticide deltamethrin, triazinone insecticide pymetrozine, and insect growth regulator buprofezin, were microinjected into RB *L. striatellus*. The relative expression of RBSDV replication related genes, *S5-1*, *S6*, and *S9-1*, and coat protein gene, *S10,* were analyzed in RB *L. striatellus* injected with the four insecticides or acetone control. All the four insecticide treatments significantly decreased the relative expression of RBSDV *S5-1*, *S6*, *S9-1,* and *S10* in *L. striatellus* (Figure 1A–D). The expression of *S10* in deltamethrin treated *L. striatellus* decreased the most, followed by buprofezin and pyrethroid treatment, which decreased to 13.67%, 22.33%, and 29.33% of the control. The expression of *S10* in the *L. striatellus* treated with imidacloprid decreased the least. These results showed that these four insecticides inhibited virus abundance in *L. striatellus*.

### 3.2. Inhibition of P450s Promotes RBSDV Infection in L. striatellus

**Figure 2:**
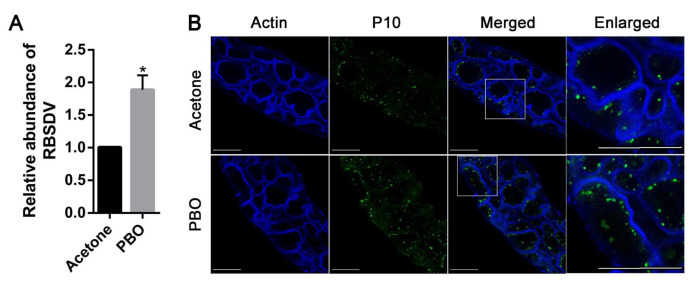
The effects of piperonyl butoxide (PBO) on RBSDV infection in its insect vector, *L. striatellus*. (**A**) RBSDV-infected *L. striatellus* was treated with PBO or acetone and, then, reared on rice seedlings for three days. The relative abundance of RBSDV was analyzed by calculating the expression of RBSDV *S10* using RT-qPCR. The asterisk (*) indicates *p* < 0.05. (**B**) Midguts of *L. striatellus* treated with PBO or acetone were dissected, and RBSDV in the midgut was labeled by immunofluorescence analysis using antibodies raised against the outer capsid protein P10 of RBSDV. The cytoskeleton actin was labeled with phalloidin. The enlarged image shows the area in the square of the merged images. Scale bars, 50 μm. Actin representing the cytoskeleton; P10 representing the outer capsid protein of RBSDV; merged representing the composite of Actin and P10; enlarged representing the zoom of the highlighted square area of the merged image.

The application of imidacloprid, deltamethrin, pymetrozine, and buprofezin could result in the overexpression or induction of P450s in planthoppers [14,24,25]. To analyze whether P450s regulate virus infection in insects, RB *L. striatellus* was injected with PBO, an inhibitor of P450s and acetone as control. After rearing on healthy rice seedlings for another three days, the RBSDV abundance was analyzed by calculating the relative expression of RBSDV *S10* using RT-qPCR. The results showed that the relative abundance of RBSDV was significantly increased after PBO treatment (Figure 2A). Additionally, the midgut of *L. striatellus* injected with either PBO or acetone were dissected, and RBSDV was labeled by immunofluorescence analysis using its specific antibody. The fluorescence dots in the midgut of *L. striatellus* treated with PBO are much more than that in the acetone control (Figure 2B). These data demonstrate that inhibition of P450s promote RBSDV abundance in *L. striatellus*.

### 3.3. Transcriptional Response of Thirty-Five P450s under RBSDV Infection

**Figure 3:**
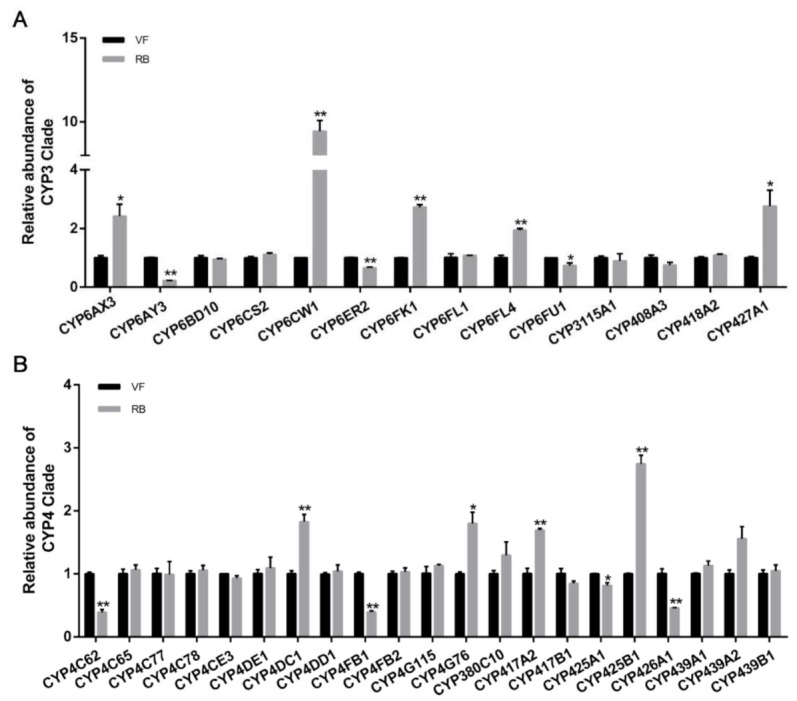
The expression of thirty-five P450s in CYP3 clade and CYP4 in *L. striatellus* under RBSDV infection. (**A**) Analysis of expression of P450s in CYP3 clade by RT-qPCR. The 3rd-instar nymphs of RBSDV-free *L. striatellus* were reared on RBSDV-infected plants and transferred to healthy rice seedlings until 5th-instar nymphs. The *L. striatellus* was collected as RBSDV-infected *L. striatellus*, and the nymphs reared on the healthy rice seedlings were collected as control. (**B**) Analysis of the expression of P450s in CYP4 clade by RT-qPCR. The asterisk (*) and asterisks (**) indicate *p* < 0.05 and *p* < 0.01.

To determine the specific P450s that might be involved in RBSDV infection, fourteen P450 genes in the CYP3 clade and twenty-one genes in the CYP4 clade were identified in the genome of *L. striatellus* (Table S3). The relative expression levels of the thirty-five P450s were compared in RB and VF populations of *L. striatellus* using RT-qPCR. Among the thirty-five P450s, sixteen P450s were significantly differently expressed after RBSDV infection, including nine upregulated and seven downregulated P450s (Figure 3). Among the fourteen P450 genes in CYP3 clade, five were upregulated and three were downregulated (Figure 3A). Notably, the expression level of *CYP6CW1* increased the most—upregulated more than 9.0-fold—while *CYP6AY3* decreased to 25% of the control (Figure 3A).

### 3.4. The Expression Profiles of CYP6CW1 and CYP6AY3 in L. striatellus

**Figure 4:**
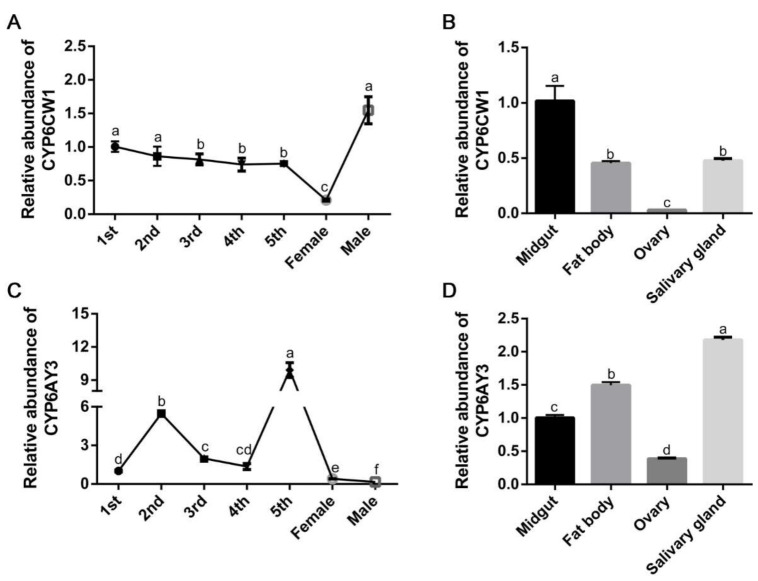
The expressions of *CYP6CW1* and *CYP6AY3* in different developmental stages and different tissues of *L. striatellus*. (**A**,**B**) Analysis of *CYP6CW1* expression at developmental stages (**A**) and different tissues (**B**) by RT-qPCR. VF *L. striatellus* from 1st-instar to adult were collected as samples at developmental stages. The fat body, midgut, ovary, and salivary gland of *L. striatellus* were dissected from VF adults of *L. striatellus* under microscope. (**C**,**D**) Analysis of *CYP6AY3* expression at developmental stages (**C**) and different tissues (**D**). The letters a, b, c, d, e, and f represent significant differences (*p* < 0.05). The conditions within a graph that share the same letters were not significantly different.

To further study the expression profiles of *CYP6CW1* and *CYP6AY3* in *L. striatellus*, the expressions of *CYP6CW1* and *CYP6AY3* were further examined in different tissues and at different developmental stages in VF *L. striatellus*. The fat body, midgut, ovary, and salivary gland of *L. striatellus* were dissected under microscope and the *L. striatellus* at developmental stages from 1st-instar to adult were collected. The relative expressions of *CYP6CW1* and *CYP6AY3* were analyzed by RT-qPCR. As shown in Figure 4A, *CYP6CW1* was expressed at all developmental stages, with the highest expression in males and the lowest in females. The expression of *CYP6CW1* in different tissues revealed that *CYP6CW1* had the highest expression in the midgut and the lowest expression in the ovary (Figure 4B). Different from the expression pattern of *CYP6CW1* at different developmental stages, *CYP6AY3* had the highest expression in the 5th-instar nymph and the lowest expression in the male (Figure 4C). The expression of *CYP6AY3* in different tissues showed that it was highly expressed in the salivary gland (Figure 4D). Similar to *CYP6CW1*, *CYP6AY3* had the lowest expression in the ovary (Figure 4D). 

### 3.5. Knockdown of CYP6CW1 and CYP6AY3 Promotes RBSDV Infection

**Figure 5:**
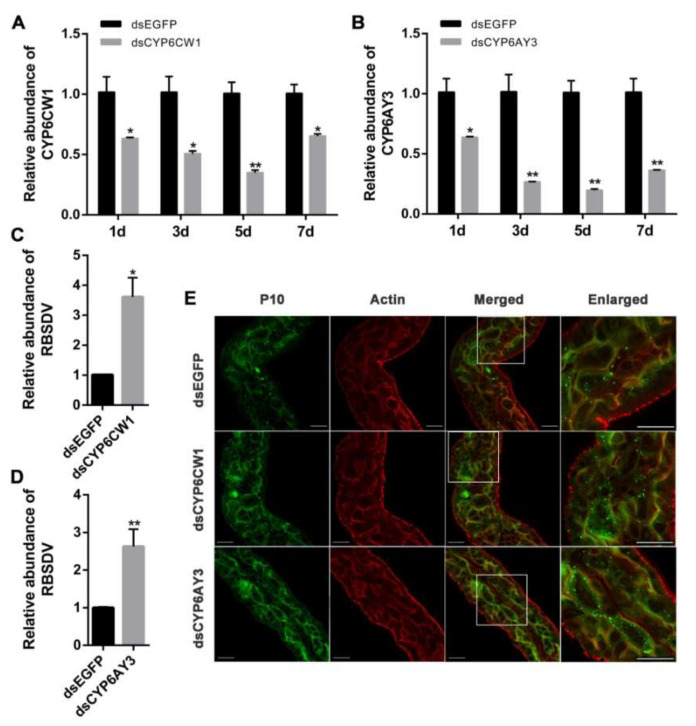
Knockdown of *CYP6CW1* and *CYP6AY3* increased RBSDV abundance in *L. striatellus*. (**A**,**B**) The dsRNAs were injected into VF *L. striatellus*. One, three, five, and seven days after dsRNA injection, the expression level of *CYP6CW1* (**A**) and *CYP6AY3* (**B**) were analyzed by RT-qPCR. (**C**,**D**) *CYP6CW1* and *CYP6AY3* were knocked down by injecting dsRNA into RBSDV-infected *L. striatellus*. *L. striatellus* injected with ds*EGFP* were included as a control. The virus abundance in RBSDV-infected *L. striatellus* injected with ds*CYP6CW1* (**C**) and ds*CYP6AY3* (**D**) was compared with that in control injected with ds*EGFP* by RT-qPCR. (**E**) RBSDV-infected *L. striatellus* was injected with dsRNAs and reared on healthy rice seedlings for three days. The virus in the midgut was labeled by immunofluorescence, and the cytoskeleton actin was labeled with phalloidin. The enlarged image shows the area in the square of the merged images. Scale bars, 50 μm. The asterisk (*) and asterisks (**) indicate *p* < 0.05 and *p* < 0.01. Actin representing the cytoskeleton; P10 representing the outer capsid protein of RBSDV; merged representing the composite of Actin and P10; enlarged representing the magnification of the selected area of the merged image.

The midgut is a barrier for RBSDV infection in its insect vector, while the salivary gland is critical for virus release. The relatively high expression of *CYP6CW1* in the midgut and *CYP6AY3* in salivary gland suggested that these two genes may be involved in regulation of virus in *L. striatellus*. To analyze whether *CYP6CW1* and *CYP6AY3* regulate RBSDV abundance in *L. striatellus*, we knocked down the expression of *CYP6CW1* and *CYP6AY3* by RNAi assay. The RB *L. striatellus* was injected with ds*CYP6CW1* and ds*CYP6AY3*. RNAi efficiency analysis showed that the relative expression levels of *CYP6CW1* and *CYP6AY3* were significantly decreased after one, three, five, and seven days of dsRNA injection (Figure 5A,B). Knockdown of *CYP6CW1* and *CYP6AY3* significantly increased the relative expression of RBSDV *S10* in *L. striatellus* (Figure 5C,D). The fluorescence dots in RB *L. striatellus* injected with ds*CYP6CW1* or ds*CYP6AY3* were much more than that in the control (Figure 5E). These results revealed that knockdown of *CYP6CW1* and *CYP6AY3* increased the RBSDV abundance in *L. striatellus*. 

### 3.6. CYP6CW1 and CYP6AY3 Negatively Regulates the Replication of Viruses

**Figure 6:**
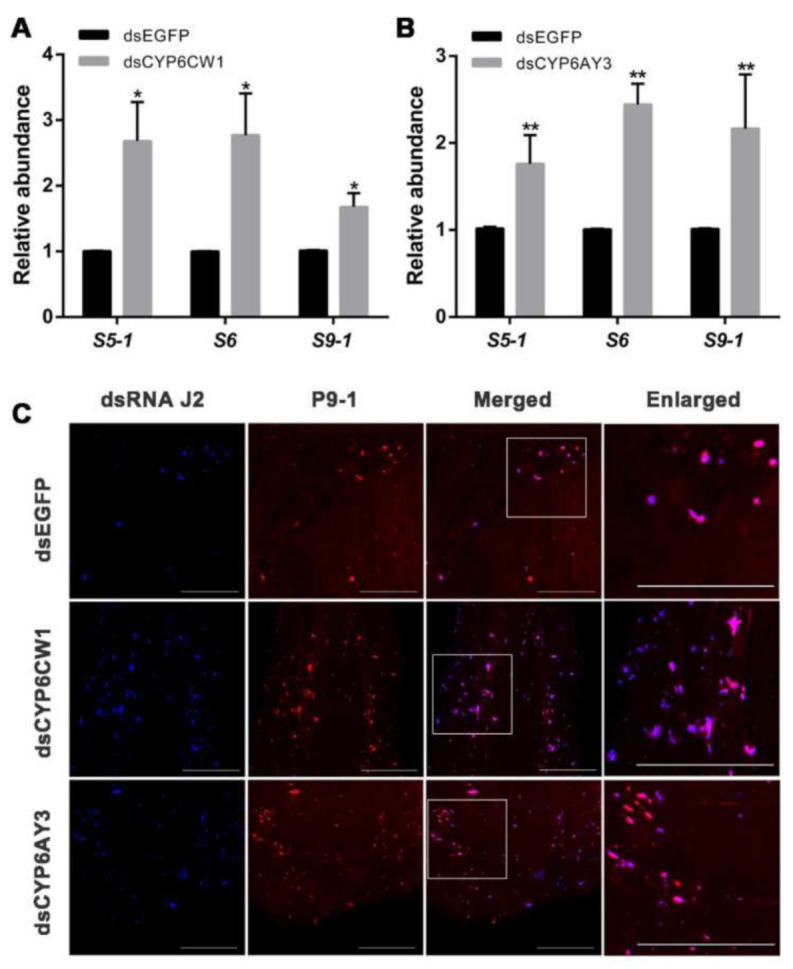
Knockdown of *CYP6CW1* and *CYP6AY3* increased RBSDV replication in *L. striatellus*. (**A**,**B**) RBSDV-infected *L. striatellus* were injected with the dsRNAs. The expression level of RBSDV *S5-1*, *S6,* and *S9-1* in *L. striatellus* knocked down of *CYP6CW1* (**A**) and *CYP6AY3* (**B**) were analyzed by RT-qPCR three days after injection. (**C**) The P9-1 protein and dsRNA, which are involved in virus replication, were labeled in the *L. striatellus* knocked down of *CYP6CW1* and *CYP6AY3* using P9-1 and dsRNA J2 antibody by immunofluorescence analysis. The asterisk (*) and asterisks (**) indicate *p* < 0.05 and *p* < 0.01. Scale bars, 50 μm.

We then sought to determine whether *CYP6CW1* and *CYP6AY3* affect virus infection by regulating virus replication in its insect vector. The *CYP6CW1* and *CYP6AY3* in RB *L. striatellus* were knocked down by injection of dsRNAs. Three days after injection, the expression levels of RBSDV replication-related genes *S5-1*, *S6,* and *S9-1* were compared with that in control injected with ds*EGFP* by RT-qPCR. As shown in Figure 6A,B, the expression levels of RBSDV *S5-1*, *S6,* and *S9-1* were significantly upregulated in the *L. striatellus* knocked down lines of *CYP6CW1* and *CYP6AY3*. 

Viroplasm is required for virus replication and dsRNAs are produced during replicative cycle. RBSDV P9-1 is the component of the virus viroplasm, and the dsRNA could be labeled as a marker for virus replication [17]. We labeled P9-1 protein and the dsRNA with an anti-dsRNA (J2) antibody in the midgut of *L. striatellus* injected with ds*CYP6CW1*, ds*CYP6AY3,* and ds*EGFP* by immunofluorescence. The fluorescence dots of J2 and P9-1 protein in the midgut of *L. striatellus* injected with ds*CYP6CW1* and ds*CYP6AY3* were much more than that in the control (Figure 6C). Taken together, these results demonstrate that knockdown of *CYP6CW1* and *CYP6AY3* promotes RBSDV replication in *L. striatellus*. 

## 4. Discussion

Most plant viral diseases are transmitted by insect vectors in the field. *Laodelphax striatellus* is a destructive agricultural pest with a wide distribution all over the world and causes the occurrence and prevalence of multiple plant viral diseases [17]. Insecticides are commonly used to control the *L. striatellus* population in the field. Little is known about the effects of insecticide application on virus infection in *L. striatellus*. 

A previous study showed that application of flupyradifurone and acetamiprid, two neonicotinoid insecticides, in potato could significantly inhibit *Bemisia tabaci* to transmit tomato chlorosis virus (ToCV) and disrupt feeding behavior of *B. tabaci* [28]. Conversely, the propagation and transmission efficiency of tomato spotted wilt orthotospovirus (TSWV) were significantly increased in Spinosad-resistant western flower thrips, *Frankliniella occidentalis* [29]. It needs to be studied whether the insecticides used to control the planthoppers can also inhibit the transmission of plant virus. In this study, we selected four representative insecticides, including imidacloprid, deltamethrin, pymetrozine, and buprofezin, to study whether insecticides could affect RBSDV infection in *L. striatellus*. The results in the present study demonstrated that application of four types of insecticides caused a significantly decrease in RBSDV abundance in *L. striatellus*. The result was in keeping with the finding observed in *B. tabaci*, whose insecticide application reduced virus infection in insect vector [28]. Imidacloprid and deltamethrin are insecticides that act on neural targets. A previous study has reported that the neural factor *Hikaru genki* homolog of the leafhopper *Nephotettix cincticeps* restrained the spread of the nucleorhabdovirus rice yellow stunt virus (RYSV) in the central nervous system of vector [30]. We speculated that application of imidacloprid and deltamethrin may reduce RBSDV infection by interfering with nerve signal transmission. In addition, imidacloprid can be used as a seed-treatment insecticide, and imidacloprid treatment can decrease the barley yellow dwarf virus-infected plants and increase the yield of the virus-infected or -uninfected susceptible oat cultivar [31]. Pymetrozine is a selective inhibitor of the feeding of sap-sucking insects, and buprofezin can interfere with metabolism and inhibit chitin synthesis in insects. It was possible that pymetrozine application reduced RBSDV infection by influencing *L. striatellus*’ feeding behavior, and buprofezin may reduce RBSDV infection by influencing *L. striatellus*’ metabolic system. These studies suggested that insecticides have different effects on different viruses in insects. These insecticides have multiple properties such as high efficiency, low toxicity, and low residue. These four types of insecticides are most widely used to control planthoppers in the fields, and insecticides should be used interchangeably. Suitable insecticides were selected according to the resistance and virulence of pests in the field. 

P450s are divided into CYP2, CYP3, CYP4, and mitochondrial CYP clades. They play critical roles in xenobiotic metabolism and insecticide resistance in insects, especially CYP3 and CYP4 clades. For instance, CYP3 and CYP4 clades played positive roles in neonicotinoid insecticide resistance in *Nilaparvata lugens* [11]. Furthermore, the application of insecticides can regulate the expression of P450s [14,24,25]. Currently, the association between P450s and insect-infecting viruses is less known. Our study is the first case to reveal that P450s regulate virus abundance and replication in insects. PBO is an effective inhibitor of P450s, which can inhibit the activity of cytochrome P450 enzymes [25]. The abundance of RBSDV was significantly increased in *L. striatellus* after PBO treatment, indicating that P450s are involved in regulating virus infection in *L. striatellus*. 

Although P450s in many insects were already identified, they were not systematically analyzed in *L. striatellus* [25,32,33]. Similar to other planthoppers, we identified 14 P450s in CYP3 clade and 21 in CYP4 clade in *L. striatellus*. Among them, sixteen P450s were significantly changed after virus infection. Similarly, a previous study has shown that the relative expression levels of detoxification enzyme genes were altered after plant virus infection in *B. tabaci* [34]. Our results indicate that P450s are response to RBSDV infection. *CYP6CW1* was the gene that increased the most, while *CYP6AY3* decreased the most after RBSDV infection in *L. striatellus*. Knockdown of *CYP6CW1* and *CYP6AY3* could increase the RBSDV abundance in *L. striatellus*, indicating that these two P450 genes are required for insects resisting virus infection. How these two P450 genes regulate virus infection remains to be investigated. *L. striatellus* might upregulate the expression of *CYP6CW1* to suppress RBSDV infection, while *CYP6AY3* expression was downregulated by RBSDV to promote virus infection. In addition to *CYP6CW1* and *CYP6AY3*, there are another five P450 genes in the CYP3 clade and eight P450s in the CYP4 clade that are responsive to RBSDV infection; these genes may also be involved in the regulation of virus infection in *L. striatellus*.

The midgut in an insect vector is an infection barrier and the salivary gland is a release barrier for the persistent virus transmitted by the insect [16]. The different expression patterns of *CYP6CW1* and *CYP6AY3* suggest they have different roles during virus infection in insects. The high expressions of *CYP6CW1* in the midgut and in RB *L. striatellus* suggest that *CYP6CW1* may play a critical role in the inhibition of virus infection in the midgut. *CYP6AY3* is downregulated after RBSDV infection, indicating that RBSDV suppresses the expression of *CYP6AY3* to facilitate its infection in *L. striatellus*. The low expression of *CYP6CW1* in females and *CYP6AY3* in males and females suggest that they may facilitate virus infection in adults. Opposite to the expression pattern of *CYP6AY3*, *CYP6CW1* had the highest expression level in male. The high expression level of *CYP6CW1* may enhance the ability to metabolize insecticides and to inhibit virus infection in male of *L. striatellus*. In insects, the high expression level of *CYP6CW1* and *CYP6AY3* was reported to associate with insecticides metabolism in resistant strains, and insecticides treatment could upregulate the expression of *CYP6CW1* and *CYP6AY3* [11]. *CYP6CW1* was related to buprofezin resistance in a lab-selected resistant strain, and *CYP6AY3v2* can hydroxylate imidacloprid in *L. striatellus* [10,35]. RBSDV infection can regulate P450s expression and may change the susceptibility of *L. striatellus* to insecticides. 

Taken together, our results revealed the effect of insecticides and P450s on virus infection in insects. Application of four commonly used insecticides inhibits RBSDV abundance in *L. striatellus*. Treatment of P450s inhibitor PBO reduced the abundance of RBSDV in *L. striatellus*. The P450 genes in CYP3 clade and CYP4 clade were systematically identified. *CYP6CW1* and *CYP6AY3* in P450s CYP3 clade were differentially expressed under RBSDV infection and were required for RBSDV replication in *L. striatellus*. These results provide new understanding the effects of P450 genes on virus infection in the insect vector and provide a theoretical basis for guiding the control of plant virus disease in the field.

## Data Availability

The data supporting the findings of this study are available within the article and Supplementary Materials.

## Supplementary Materials

The following are available online at https://www.mdpi.com/article/10.3390/v13081576/s1, Table S1: Primers used in this study, Table S2: ANOVA Fs, Table S3: Summary of the fourteen CYP3 clade and twenty-one CYP4 clade P450 genes identified from L. striatellus genome.
