# Supplementary material for: Cytochrome P450 Monooxygenases CYP6AY3 and CYP6CW1 Regulate Rice Black-Streaked Dwarf Virus Replication in Laodelphax striatellus (Fallén)
Source: Viruses. 2021 Aug 10;13(8):1576. doi: 10.3390/v13081576 (PMC8402780; doi:10.3390/v13081576)
Supplement: Supplementary file 1 [file viruses-13-01576-s001.zip › Table S3.pdf]

Table S3. Summary of the fourteen CYP3 clade and twenty-one CYP4 clade P450 genes identified from *L. striatellus* genome.

Clade	Gene ID	Name	ORF (bp)	Species	Acc. number	Score	E-value
CYP3	Contig196.5	CYP6AX3	1542	*Sogatella furcifera*	AQM57042.1	941	0.00
	Contig101.2	CYP6AY3	1509	*Laodelphax striatellus*	AFU86482.1	1033	0.00
	Contig166.61	CYP6BD10	1560	*Laodelphax striatellus*	AFU86445.1	1044	0.00
	Contig8.263	CYP6CS2	1542	*Laodelphax striatellus*	AFU86422.1	984	0.00
	Contig417.17	CYP6CW1	3384	*Nilaparvata lugens*	AIW79985.1	865	0.00
	Contig686.5	CYP6ER2	1536	*Laodelphax striatellus*	AFU86438.1	810	0.00
	Contig8.262	CYP6FK1	1533	*Laodelphax striatellus*	AFU86431.1	978	0.00
	Contig1140.1	CYP6FL1	1503	*Sogatella furcifera*	AQM57060.1	822	0.00
	Contig376.3	CYP6FL4	1590	*Nilaparvata lugens*	AIW79988.1	864	0.00
	Contig615.20	CYP6FU1	1446	*Laodelphax striatellus*	AFU86479.2	697	0.00
	Contig51.36	CYP3115A1	1428	*Laodelphax striatellus*	RZF42923.1	892	0.00
	Contig615.5	CYP408A3	1548	*Sogatella furcifera*	AQM57048.1	875	0.00
	Contig457.18	CYP418A2	1536	*Laodelphax striatellus*	AFU86461.1	1022	0.00
	Contig457.19	CYP427A1	1512	*Nilaparvata lugens*	AIW79993.1	888	0.00
CYP4	Contig8.294	CYP4C62	1563	*Sogatella furcifera*	AQM57036.1	1040	0.00
	Contig222.27	CYP4C65	759	*Sogatella furcifera*	AQM57054.1	468	5.00E-162
	Contig222.28	CYP4C77	3834	*Nilaparvata lugens*	AIW79997.1	901	0.00
	Contig741.11	CYP4C78	1575	*Nilaparvata lugens*	AIW79999.1	816	0.00
	Contig286.5	CYP4CE3	1620	*Sogatella furcifera*	AQM57037.1	960	0.00
	Contig113.54	CYP4DE1	1590	*Nilaparvata lugens*	AIW80004.1	1043	0.00
	Contig522.9	CYP4DC1	1509	*Sogatella furcifera*	AQM57056.1	898	0.00
	Contig26.134	CYP4DD1	1572	*Laodelphax striatellus*	AFU86473.1	456	4.00E-157
	Contig158.38	CYP4FB1	1602	*Laodelphax striatellus*	AYE67169.1	968	0.00
	Contig353.29	CYP4FB2	1524	*Laodelphax striatellus*	RZF39244.1	1021	0.00
	Contig318.10	CYP4G115	1731	*Nilaparvata lugens*	AIW80009.1	974	0.00
	Contig510.12	CYP4G76	1191	*Sogatella furcifera*	AQM57059.1	645	0.00
	Contig112.33	CYP380C10	1644	*Nilaparvata lugens*	AIW79998.1	993	0.00
	Contig372.7	CYP417A2	1524	*Nilaparvata lugens*	AIW80013.1	1024	0.00
	Contig372.25	CYP417B1	1470	*Laodelphax striatellus*	AFU86448.1	979	0.00
	Contig13.305	CYP425A1	1539	*Laodelphax striatellus*	AFU86443.1	1055	0.00
	Contig13.306	CYP425B1	1569	*Nilaparvata lugens*	AIW80020.1	919	0.00
	Contig348.20	CYP426A1	1494	*Laodelphax striatellus*	AFU86430.1	1029	0.00
	Contig31.178	CYP439A1	1470	*Laodelphax striatellus*	AFU86477.1	990	0.00
	Contig88.25	CYP439A2	1494	*Laodelphax striatellus*	RZF35586.1	1033	0.00
	Contig1477.1	CYP439B1	801	*Laodelphax striatellus*	RZF49230.1	546	0.00
